# Genetic models of fibrillinopathies

**DOI:** 10.1093/genetics/iyad189

**Published:** 2023-11-16

**Authors:** Kim M Summers

**Affiliations:** Mater Research Institute-University of Queensland, Translational Research Institute, Woolloongabba QLD 4102, Australia

**Keywords:** fibrillin-1, fibrillin-2, fibrillin-3, Marfan syndrome, congenital contractural arachnodactyly, fibrillinopathies

## Abstract

The fibrillinopathies represent a group of diseases in which the 10–12 nm extracellular microfibrils are disrupted by genetic variants in one of the genes encoding fibrillin molecules, large glycoproteins of the extracellular matrix. The best-known fibrillinopathy is Marfan syndrome, an autosomal dominant condition affecting the cardiovascular, ocular, skeletal, and other systems, with a prevalence of around 1 in 3,000 across all ethnic groups. It is caused by variants of the *FBN1* gene, encoding fibrillin-1, which interacts with elastin to provide strength and elasticity to connective tissues. A number of mouse models have been created in an attempt to replicate the human phenotype, although all have limitations. There are also natural bovine models and engineered models in pig and rabbit. Variants in *FBN2* encoding fibrillin-2 cause congenital contractural arachnodactyly and mouse models for this condition have also been produced. In most animals, including birds, reptiles, and amphibians, there is a third fibrillin, fibrillin-3 (*FBN3* gene) for which the creation of models has been difficult as the gene is degenerate and nonfunctional in mice and rats. Other eukaryotes such as the nematode *C. elegans* and zebrafish *D. rerio* have a gene with some homology to fibrillins and models have been used to discover more about the function of this family of proteins. This review looks at the phenotype, inheritance, and relevance of the various animal models for the different fibrillinopathies.

## Teaser

Fibrillinopathies are a group of connective tissue disorders in humans that affect the bones, cardiovascular system, and eyes. To further our understanding of these disorders, they have been modelled in mice, cattle, rabbits, pigs, zebrafish, and some non-vertebrate animals, with varying degrees of success in replicating the human disease phenotypes. Here, Summers reviews the existing fibrillinopathy models, including their phenotypes, mode of inheritance, and relevance to the human disease in question, and discusses other novel models that might help in understanding these conditions

## Introduction

This review considers a range of small and large animal models for diseases caused by variants in the fibrillin gene family and future directions that may be profitable. Animal models serve a number of purposes in attempts to understand the etiology of human genetic disease. First, they can help elucidate the mechanisms by which genes function in normal and pathological situations. Second, they can provide a tool for testing pharmacological approaches to treatment or prevention. They can also be useful for developing other treatment strategies such as surgery and physical therapy. Genome editing techniques using clustered regularly interspaced short palindromic repeats (CRISPR) with the Cas9 enzyme, zinc finger nucleases, and transcription activator-like effector nucleases (TALENs) (Gaj et al. 2016) have opened the possibility of precisely engineering genetic changes in both laboratory animals and other less conventional model species (Whitelaw et al. 2016). However, most models are still created in the mouse, usually in the C57BL/6 strain, which has characteristics atypical of other mouse strains (Orozco et al. 2009; Keane et al. 2011; Lilue et al. 2018; Hume 2023) and other mammals. Mice are very different from humans, in size, metabolism, physiology, anatomy, and life history (Perlman 2016). Drugs that appear to be helpful in the mouse model frequently fail human trials (for example, anticancer drugs) (Anisimov et al. 2005; De Jong and Maina 2010; Adams 2012), and the small size of the mouse makes testing of some approaches, for example surgical treatments, difficult.

The fibrillin gene family encodes 3 large glycoproteins of the extracellular matrix in humans (Dietz et al. 1991; Lee et al. 1991; Maslen et al. 1991; Nagase et al. 2001; Corson et al. 2004) (reviewed in Davis and Summers 2012; Summers et al. 2023). Phylogenetic analysis indicates that fibrillins probably arose via duplication of an ancestral gene before divergence of the ray-finned fishes. One copy evolved to encode fibrillin-1, while the other copy was further duplicated at the time of the divergence of the tetrapods, to encode fibrillin-2 and fibrillin-3 (Robertson et al. 2011; Piha-Gossack et al. 2012). Three fibrillin genes have been identified in almost all mammals, birds, amphibians, reptiles, and fish that have been sequenced, with the exception of mice and rats (2 fibrillin genes) and some fish (1 or 2 fibrillin genes). The fibrillins have a characteristic structure comprising multiple epidermal growth factor-like (EGF) domains interspersed with several transforming growth factor beta (TGFB) binding protein domains (TB domains) and hybrid domains which appear to have resulted from the fusion of an EGF domain with a TB domain (Piha-Gossack et al. 2012; Kielty 2017; Summers et al. 2023). While many proteins have multiple EGF domains, only fibrillins and latent TGFB binding proteins (LTBPs) contain the TB and hybrid domains. It has been suggested that only proteins with these unique characteristics should be considered fibrillin homologs (Piha-Gossack et al. 2012).

Fibrillins form 10–12 nm microfibrils which interact with elastin in connective tissues (Kielty 2017; Zhang et al. 2022). The microfibrils can also exist in the absence of elastin, for example in the ciliary zonule fibers that hold the lens of the eye in place (Bassnett 2021). Fibrillins interact with the LTBPs to regulate the bioavailability of TGFB family members (Isogai et al. 2003; Hirani et al. 2007; Massam-Wu et al. 2010). Diseases resulting from a deficiency of fibrillins have been termed fibrillinopathies and generally affect the extracellular matrix of connective tissues in humans. In this paper, the animal models of fibrillinopathies are reviewed with consideration of whether they faithfully reproduce the human condition. Mouse models of fibrillinopathies do not entirely replicate the human phenotypes (Miller et al. 2010). Models in large animals may be more appropriate but are expensive to generate and maintain (Whitelaw et al. 2016). Here the relevance of mouse models is addressed and potential models in other species are described.

## Fibrillin-1 and Marfan syndrome

The best-studied fibrillin family member is fibrillin-1 (human *FBN1* gene, chromosome 15). Fibrillin-1 is generally associated with the extracellular matrix of connective tissues including bone, muscle, and adipose in adults (Summers et al. 2010) and is also the major component of the adult ciliary zonule fibers (Streeten et al. 1981; Streeten et al. 1983; Mayne et al. 1991; Wright et al. 1994; Bassnett 2021). Consistent with these structural roles, *FBN1* variants are associated with cardiovascular, ocular, skeletal, and other phenotypic effects (Loeys et al. 2010). Pathogenic variants of *FBN1* usually cause the phenotype of Marfan syndrome (MFS) (Table 1) (Pyeritz 2000; Robinson and Godfrey 2000; Collod-Beroud et al. 2003; Faivre et al. 2007; Faivre et al. 2009; Collod-Beroud et al. 2014), the most common fibrillinopathy. The major cause of mortality in MFS is aortic dilatation and dissection, but considerable physical and psychosocial morbidity arises from aortic valve dysfunction, lens subluxation, respiratory issues, and skeletal deformities such as scoliosis and excessive height (Velvin et al. 2015). A rapidly progressing early onset form of MFS results from altered amino acids within the central portion of the protein (Faivre et al. 2009; Collod-Beroud et al. 2014).

**Table 1.** Fibrillin genes and diseases in humans.

Gene	OMIM number of gene	Position	Condition	OMIM number of condition	Phenotype	Inheritance
FBN1	134797	15:48,408,313–48,645,709	Acromicric dysplasia	102370	Short stature, skin thickening, joint limitations	Dominant
			Ectopia lentis, familial	129600	Subluxation and dislocation of the optical lens, without other Marfanoid features	Dominant
			Geleophysic dysplasia 2	614185	Short stature, skin thickening, joint limitations	Dominant
			Marfanoid-progeroid-lipodystrophy syndrome (also known as neonatal progeroid syndrome)	616914	Low or absent subcutaneous fat, with or without other Marfanoid features	Dominant
			MFS	154700	Aortic dilatation and dissection, ectopia lentis, overgrowth of the long bones usually with tall stature, other connective tissue abnormalities	Dominant
			MASS syndrome	604308	Involvement of mitral valve, aorta, skeleton, skin	Dominant
			Stiff-skin syndrome	184900	Hard thick skin	Dominant
			WMS2, dominant	608328	Short stature, brachydactyly, joint stiffness, lens abnormalities	Dominant
FBN2	612570	5:128,257,909–128,538,245	Contractural arachnodactyly, congenital	121050	Arachnodactyly, flexion contractures of multiple joints, kyphoscoliosis, “crumpled” ears, Marfanoid habitus	Dominant
			Macular degeneration, early onset	616118	Atrophic macular disease in fifth decade without skeletal features	Dominant
FBN3	608529	19:8,065,402–8,149,592	Polycystic ovary syndrome 1 (tentative association)	184700	Amenorrhea, obesity, hirsutism	Polygenic

Information compiled from On Line Mendelian Inheritance in Man (OMIM; https://omim.org/), GeneReviews (https://www.ncbi.nlm.nih.gov/) and references therein.

An ideal model of MFS would replicate the phenotype and inheritance pattern of the human condition. Specifically, the phenotype should be inherited in a dominant fashion, with high penetrance but variable expressivity, even among individuals carrying the same variant. The 2 cardinal signs of MFS, aortic dilatation/dissection and lens subluxation (Loeys et al. 2010), should be present. Ideally, the model would also show similar skeletal anomalies, for example disproportionately long limbs and scoliosis and potentially the lung anomalies. As with the human disease, intrafamilial variability would be expected at least in outbred animals. Mouse models to date have lacked some of these features, although large animal models are closer in phenotype.

### Mouse models of MFS

The first mouse model for MFS, mgΔ, was created in 1997 (Pereira et al. 1997). Here a DNA stretch of 6 kb within the *Fbn1* gene was replaced with a neomycin resistance cassette, resulting in the deletion of 272 residues in the equivalent region to that involved in the human early onset disease (the “neonatal region”). Homozygous animals showed reduction in *Fbn1* mRNA and extracellular fibrillin-1 protein to about 10% of wild type (Pereira et al. 1997). There were no gross phenotypic abnormalities at birth, but all died suddenly of cardiovascular complications before 3 weeks of age. Heterozygote mgΔ/+ mice were indistinguishable from wild-type littermates, hence this model does not replicate the dominant inheritance pattern of the human condition, although the homozygotes showed similar cardiovascular pathology to human heterozygotes.

Other models have presented additional features of MFS (Table 2). The mgR model (Pereira et al. 1999) showed a reduction of fibrillin-1 to approximately 20% of wild type in skin and lung and variable reductions in other tissues. The phenotype of homozygotes included late onset vascular and skeletal abnormalities but heterozygotes showed no effects.

**Table 2.** Mouse models of fibrillin-1 syndromes.

Model name	Variant type	Strain	Phenotype	Inheritance	Syndrome	References
mgΔ	in frame del 6 kb ins PGK*neo*	C57BL/6J	Aortic dilatation, pre weaning lethal	recessive	MFS	Pereira et al. (1997)
mgR	ins PGK*neo*	C56BL/6	Vascular abnormalities, kyphosis, bone overgrowth	recessive	MFS	Pereira et al. (1999)
mgN	del 700 bp ins PGK*neo*	C57BL/6J	Aortic aneurysm, impaired respiratory function, pre weaning lethal	recessive	MFS	Carta et al. (2006)
mgΔloxPneo	in frame del exons 19–24 ins PGK*neo* ins 2 × loxP	C57BL/6	Vascular abnormalities and skeletal phenotype by 9 months, severe pulmonary alterations by 6 months, ocular abnormalities including ectopia lentis, homozygote lethal pre E13	dominant	MFS	Lima et al. (2010), de Souza et al. (2019), and Souza et al. (2021)
		129/Sv	Vascular abnormalities, severe pulmonary alterations, skeletal phenotype by 3 months, interindividual variability, homozygote lethal pre E13	dominant	MFS	
		Mixed 129/Sv and CD-1	Sporadic spinal deformities	dominant		
GT-8	truncation after exon 32 ins GFP	C57BL/6	Abnormal microfibril morphology, homozygote postnatal lethal P9-P18	dominant	MFS	Charbonneau et al. (2010a)
C1041G*^a^*	missense	C57BL/6J	Deterioration of aortic structure with age, skeletal anomalies, widening of airspaces, homozygote postnatal lethal P7-P10 from aortic dissection	dominant	MFS	Judge et al. (2004)
D1545E	missense	Mixed C57BL/6J and 129/SvEv	Pathological skin fibrosis, homozygote lethal pre E10.5	dominant	SSS	Gerber et al. (2013)
W1572C	missense	Mixed C57BL/6J and 129/SvEv	Pathological skin fibrosis, homozygote viable with accelerated skin fibrosis	dominant	SSS	Gerber et al. (2013)
Tsk	in frame dup 30–40 kb	B10.D2(58N)/Sn	Thick skin, visceral fibrosis, increased skeletal size, homozygote lethal pre E8	dominant	SSS	Siracusa et al. (1996)
WMΔ	del exon 10–12	C57BL/6	Thick skin, brachydactyly, early reduced long bone growth, homozygote has normal viability	dominant	WMS2	Sengle et al. (2012)
H1Δ	del exon 7, first hybrid domain	C57BL/6	Grossly normal, normal life span	no abnormal phenotype		Charbonneau et al. (2010a)
NPS	del 10 bp of exon/intron 65 border	C57BL/6	Extreme leanness, reduced appetite	dominant	Marfan-progeroid-lipodystrophy syndrome	Duerrschmid et al. (2017)
Tg(WT)	YAC including the whole wild type human *FBN1*	C57BL/6J	Production of human fibrillin-1 at normal level in addition to mouse fibrillin-1	no abnormal phenotype	NA	Judge et al. (2004)
Tg(mut3)	YAC including human *FBN1* carrying p.C1663R	C57BL/6J	Production of mutant human fibrillin-1 at 2 × normal level in addition to mouse fibrillin-1	no abnormal phenotype	NA	Judge et al. (2004)

List derived from the Mouse Genome Informatics (MGI) database (http://www.informatics.jax.org/allele/summary? markerId=MGI:95489) and relevant publications. As with the human condition, the dominant phenotype (expressed in heterozygotes) is less severe than the phenotype of homozygous or biallelic genotypes. See also Charbonneau et al. (2010a) and Hubmacher and Reinhardt (2011) for earlier summaries of available mouse variants. A number of conditional mutations in the *Fbn1* locus have also been created (for example, Cook et al. 2012); these are not listed here as they do not replicate the human situation where the variant is present in all cells and tissues.

del, deletion; ins, insertion; dup, duplication; MFS, Marfan syndrome; SSS, stiff-skin syndrome; WMS, Weill-Marchesani syndrome; E8, embryonic day 8; E13, embryonic day 13; P7, postnatal day 7; P10, postnatal day 10; bp, base pairs; kb, kilobases.

^*a*^The MGI database lists C1037G and C1039G as synonyms for C1041G (https://www.informatics.jax.org/allele/MGI:3690325).

The mgΔloxPneo model (also known as mgΔlpn; Table 2) presents with a heterozygous phenotype of vascular, skeletal, and pulmonary abnormalities. When carried on the 129/Sv background the overall phenotype was more severe than on the C57BL/6 background but there was considerable interindividual variability, consistent with the human situation. On a mixed 129/Sv and CD-1 background the phenotype was mild with the main manifestation being sporadic spinal deformities (Lima et al. 2010; De Souza et al. 2019).

In the GT-8 model, fibrillin-1 is truncated after exon 32 and replaced by the green fluorescent protein (GFP) sequence. This model showed a dominant phenotype, including abnormal microfibril morphology in aorta, skin, tendon, and skeletal muscle (Charbonneau et al. 2010a). The truncated, GFP-tagged molecule was proposed to exert a dominant negative effect on stability of microfibrils, leading to fragmentation of the elastic lamellae of the aorta (Charbonneau et al. 2010a).

The major cause of mortality in MFS is aortic dilatation (potentially including aortic valve malfunction) and dissection. While the anatomy of the adult mouse and human heart is very similar, they differ in several features, including size of the atria, position in the chest cavity, heart rate, lifelong total number of cardiac cycles, and timing of developmental events (Wessels and Sedmera 2003; Krishnan et al. 2014). Relevant to MFS, the aorta and aortic valve are formed at a much earlier developmental stage in human (around 9 weeks gestation) than in mouse (from embryonic day 12.5 to 18.5, which is just before birth), although the processes involved are similar (Krishnan et al. 2014). The aortic root and ascending aorta (the primary location of aortic dilatation in MFS) were similar in mice and humans (Casteleyn et al. 2010), although the aortic diameter of the mouse is relatively smaller than that of the human and gradients of arterial stiffness were different between species (Hopper et al. 2021). Vascular elastin is stable during a mouse lifetime of 2 years (Davis 1993), but degrades over a human lifetime, with a half-life of about 70 years (Shapiro et al. 1991). Hence vascular ageing results in loss of elastic fiber integrity in humans but not in mice. Since fibrillin-1 is an essential component of the elastic fibers this difference may be important when evaluating mouse models of MFS. These results suggest that mouse models have some limitations for cardiovascular research, and may explain the failure to replicate treatment results (discussed below).

In humans, dilatation of the ascending aorta may be detectable at birth but the rate of expansion accelerates during development and adult life. Newborn mgR homozygous mice had normal vascular anatomy and architecture (Pereira et al. 1999). Homozygotes for the 3 recessive mouse models (mgN, mgΔ, and mgR in order of increasing fibrillin-1 expression; Table 2) died within the first 2 weeks, 3 weeks, or 4 months of life, respectively (Pereira et al. 1997; Pereira et al. 1999; Carta et al. 2006). Necropsy showed aneurysm of the aortic root or ascending aorta in most animals. These results indicate relatively early expansion of the aorta, similar to the human condition. Heterozygotes for the mgΔloxPneo variant on both genetic backgrounds had a normal lifespan, but showed an age-dependent increase in the thickness of the aortic media (Lima et al. 2010). Heterozygotes for the C1041G variant had a normal life span with no evidence of aortic dissection, although a progressive deterioration of the aortic media and thickening of the aortic wall was observed after 2 months of age (Judge et al. 2004). Thus the mouse models of MFS share some features with the human condition but do not replicate the cardiovascular manifestations completely.

Subluxation of the ocular lens (ectopia lentis) is a cardinal feature of MFS (Maumenee 1981; Loeys et al. 2010). The eye of the mouse is structurally different from the human. The spherical lens takes up much of the eyeball (see Figure 1A of Souza et al. 2021) and the optic nerve and retina are different from human eyes (Levkovitch-Verbin 2004). Both fibrillin-1 and fibrillin-2 were detected in the zonule of wild-type adult mice but the zonule of adult humans only contained fibrillin-1 (Beene et al. 2013; Hubmacher et al. 2014). A study of homozygous mgN/mgN (fibrillin-1 null) and mgR/mgR (fibrillin-1 reduced) mice showed an intact ciliary zonule composed of fibrillin-2 (Beene et al. 2013). Some individuals with MFS have apparently intact ciliary zonular fibers without lens dislocation (Maumenee 1981). It is not known whether fibrillin-2 might contribute to the fibers in these cases. The ocular phenotype of the mgΔloxPneo (Table 2) model has recently been examined (Souza et al. 2021). Heterozygous mice on the C57BL/6 background were found to have ectopia lentis with virtual absence of the zonular fibers. The various models can contribute to our understanding of the role of the different fibrillins in ocular development and function, although they may not precisely model the human abnormality, for example because of the involvement of fibrillin-2 in mouse but not human zonules.

Overgrowth of the long bones and ribs is a striking morphological feature of MFS (Judge and Dietz 2005) that is not always seen in mouse models, possibly due to differences between the skeleton of mice and humans. Humans are bipedal while mice are quadrupedal, shifting the transmission of body weight from 2 to 4 limbs (Ruberte et al. 2023). Organization of bone is different in mice compared to humans (Maynard and Ackert-Bicknell 2019). However, in some aspects, the mouse is more similar to human than other quadrupedal species. For example, the mouse has a well-developed clavicle, which is reduced or absent in many species (Senter and Moch 2015). Mice are predisposed to age-related bone loss (Maynard and Ackert-Bicknell 2019), which is also seen in MFS (Folkestad et al. 2020 and papers cited therein), although this happens earlier in mice than in humans. Surprisingly, the severely affected homozygous mgΔ mice did not manifest skeletal abnormalities (Pereira et al. 1997) which may reflect a distinct pathogenesis or the mice may have died before overt skeletal signs were present. In contrast both the mgN (with negligible fibrillin-1) and mgR (with 20–25% of wildtype fibrillin-1) mice exhibited skeletal malformations including kyphosis and overgrowth of the ribs (also seen in MFS) (Pereira et al. 1999; Carta et al. 2006). There was marginal increase in limb length in some mgR homozygotes (Pereira et al. 1999) but this feature was not generally reported. Heterozygotes for the mgΔloxPneo variant presented with skeletal kyphosis (Lima et al. 2010) and the C1041G heterozygotes showed kyphosis and overgrowth of the ribs (Judge et al. 2004). Evaluation of skeletal phenotype of mouse models of MFS should take into account the differences in bone biology and skeletal structure.

There have also been a number of conditional knock-out models which allow inactivation of *Fbn1* in specific tissues or developmental stages. Conditional knock-out models overcome the homozygous lethality of many *Fbn1* variants in the mouse and have been useful to elucidate the functions of fibrillin-1. For example, *Fbn1* is highly expressed by osteoblasts (Summers et al. 2010). Conditional deletion of a floxed *Fbn1* allele (*Fbn1^Lox^*) driven by *Prx1*-cre or *Osx*-cre in osteoblasts demonstrated an essential function of fibrillin-1 in bone development, through osteoblast modulation of osteoclast activity (Cook et al. 2012). The same approach was used to delete expression of fibrillin-1 in the lens and nonpigmented ciliary epithelium of the eye (Jones et al. 2019), and showed that fibrillin-1 is important in the nonpigmented ciliary epithelium but not the lens for normal eye development. There was no abnormality in heterozygous knock-out mice. The availability of “conditional ready” mice, such as the *Fbn1^Lox^* strain, will facilitate further exploration of the impact of tissue or stage-specific removal of the *Fbn1* gene, although to date there have been few studies exploiting these resources.

A transgenic mouse model was used to argue that the dominant phenotype in MFS is due to haploinsufficiency (Judge et al. 2004). The human *FBN1* gene including all promoter elements was included on a yeast artificial chromosome and introduced into mouse ESJ1 embryonic stem cells. Chimeric founder mice were backcrossed to C57BL/6 mice for several generations and progeny were shown to express human fibrillin-1 in addition to normal expression of the mouse protein. No phenotypic abnormalities were seen, even in the presence of a known human Marfan mutation in the transgene. The studies also used the C1041G mouse model, which has a missense change (p.Cys1041Gly). In this model, the abnormal protein is synthesized but lacks one of the conserved cysteine residues, potentially resulting in formation of illegitimate disulfide bonds, which might be expected to interfere with assembly of microfibrils in a dominant negative manner. However, the phenotype of the C1041G mouse model could be rescued by the human *FBN1* transgene, suggesting that the phenotype arose because of haploinsufficiency. The authors propose that the human phenotype also results from haploinsufficiency rather than a dominant negative interaction between the mutant and normal fibrils (Judge et al. 2004), which could open up therapeutic possibilities. However, this model reflects only some human variants. Other variants clearly act in a dominant negative manner (Schrijver et al. 1999; Schrijver et al. 2002; De Backer et al. 2018).

### Potential treatments derived from mouse models

Treatment of MFS currently involves pharmacological intervention to reduce the rate of dilatation of the aorta (conventionally with beta-blockers such as atenolol and propranolol), and surgical procedures to correct aortic aneurysm, heart valve malfunction, and skeletal abnormalities and to replace dislocated lenses. Mouse models are mandatory for testing new drug treatments and have been used to explore treatments for MFS based on discoveries of the function of fibrillin-1.

Much of the phenotype of MFS has been attributed to a dysregulated increase of TGFB levels (Neptune et al. 2003; Dietz et al. 2005; Nataatmadja et al. 2006; Matt et al. 2009). TGFB blockers such as losartan were successful in normalizing aortic wall architecture and slowing aortic root dilatation of mice heterozygous for the C1041G variant. In contrast, heterozygous C1041G mice undergoing conventional treatment with the beta-blocker propranolol showed no difference from placebo treated heterozygous mice (Habashi et al. 2006). In a metanalysis of 7 clinical trials with a total of 1,442 MFS patients, both TGFB blockers and beta-blockers generally reduced aortic growth, with similar effectiveness (Pitcher et al. 2022), unlike treatment of the mouse model. It is possible that the 2 treatments act on different aspects of the phenotype (Bhatt et al. 2015; Sandor et al. 2015). In some studies, there was no effect of a TGFB blocker on aortic dilatation in humans (Milleron et al. 2015). The variability of results depended in part on the nature of the variant in each individual (Franken et al. 2015) but could also be attributed to the differences in phenotype between mouse and human. This suggests that current mouse models do not faithfully replicate the human condition and therefore treatments developed based on these models may not translate to human use. Some animal model studies indicate that TGFB activation may be a healing homeostatic response to fiber damage due to the abnormal fibrillin molecules (Charbonneau et al. 2010a; Cook et al. 2015; Sengle and Sakai 2015; Kielty 2017) and therefore blocking TGFB might have undesirable effects.

Another possible treatment for at least some of the signs and symptoms of MFS comes from a recent study of homozygous mgR/mgR mice (Lau et al. 2023) administered a high-fat diet. These animals were protected from aortic aneurysm. Since many MFS patients have low body mass index (Davis et al. 2016; Summers et al. 2023), increasing the intake of fat or total energy might be a simple but effective way of ameliorating the clinical status of some human cases.

### Large animal models of MFS

A large animal model of MFS might have more in common with the human disease than the models in mice, since the physiology is likely to be more similar. Large animals have frequently been used for both surgical and pharmaceutical cardiovascular research (Tsang et al. 2016). Published MFS models are discussed below.

Bovine fibrillinopathy has occurred spontaneously in 2 cattle breeds and bovine MFS is listed with accession OMIA 000628-9913 in Online Mendelian Inheritance in Animals (OMIA; https://www.omia.org/home/). A natural MFS model was first described in 7 Limousine cross breed calves sired by a single father (Besser et al. 1990). These animals had kyphosis, joint and tendon laxity, long thin limbs, ectopia lentis, enlarged aortic root, arch and mitral valve annulus, and histological abnormalities of elastic fibers in vivo and in vitro (Besser et al. 1990; Potter et al. 1993; Potter and Besser 1994; Gigante et al. 1999). Since the bull had a normal phenotype and only a small number of his offspring developed the MFS manifestations, the authors propose dominant inheritance resulting from germline mosaicism in the bull. The variant in the affected calves was identified as c.3598G > A (p.Glu1200Lys) affecting exon 29, a calcium binding epidermal growth factor domain within the “neonatal region” in humans. The variant was heterozygous in all affected calves and found in about 19% of sperm from the sire (Singleton et al. 2005). This model more closely replicates the human phenotype and inheritance pattern than the mouse models. If bred under controlled conditions, these cattle could potentially act as models for human disease, leading to development and trials of treatment strategies.

A second potential cattle model was produced by a spontaneous *FBN1* variant in Japanese Black cattle (Hirano et al. 2012). The phenotype was described as “MFS-like”, but more closely resembles the human Marfanoid-progeroid-lipodystrophy phenotype (Table 1) and this model is discussed further in the next section.

The generation of porcine models of MFS would have great relevance to development of treatments since pig cardiovascular systems are more similar to human than other animal models and pigs have been used extensively to develop techniques for cardiac surgery (Tsang et al. 2016). *FBN1* variants have been introduced in the pig using genome editing. Our own attempt using CRISPR-Cas9 genome editing to target exon 2 of the pig *FBN1* resulted in 2 offspring with an identical homozygous 5-base pair deletion (Tsang et al. 2020). The piglets were severely compromised by hydrops fetalis, a complication not reported for MFS or the homozygous mouse models, and likely the result of off-target effects. The neonatal phenotype did not replicate human MFS and the pigs did not survive for examination of later developmental stages. Another pig model (Umeyama et al. 2016), which used zinc finger nuclease treatment targeting exon 10, was more successful. A 1-base pair deletion was created, introducing a stop codon at amino acid residue 531 (p.Glu433AsnfsX98). Heterozygous pigs showed primarily skeletal abnormalities. Some had respiratory problems and/or abnormalities of the elastic fibers of the aorta. None of the heterozygotes in the founder or subsequent generations had dilatation of the proximal aorta, aortic dissection, or ectopia lentis (Jack et al. 2022). As in many mouse models, homozygous pigs had a severe phenotype which more accurately reflected the heterozygous MFS phenotype in humans. In particular, the homozygous mutant pigs demonstrated dilatation and dissection of the ascending aorta, ectopia lentis, and lipodystrophy, and the longest survival time was 28 days. Thus this pig model does not truly replicate the inheritance pattern of MFS, since the cardinal manifestations were only seen in homozygotes. The short lifespan of such homozygotes probably precludes this model from being used to develop surgical and pharmacological strategies for treating MFS.

## Models of Marfanoid-progeroid-lipodystrophy syndrome and asprosin deficiency

One group of *FBN1* variants results in Marfanoid-progeroid-lipodystrophy syndrome (also known as neonatal progeroid syndrome; Table 1). Affected individuals have extreme leanness, poor appetite, reduced subcutaneous fat mass with the maintenance of insulin sensitivity and euglycemia (Graul-Neumann et al. 2010; Goldblatt et al. 2011; Horn and Robinson 2011; Takenouchi et al. 2013; Jacquinet et al. 2014; Romere et al. 2016; Lin et al. 2020). They lack the ability to produce the C-terminal fragment of fibrillin-1, because of disruption of a critical furin cleavage site, deletion of the C-terminal coding sequence, or premature termination of protein synthesis (reviewed by Romere et al. 2016; see also Duerrschmid et al. 2017; Summers et al. 2023). This C-terminal 140 amino acid product of the terminal exons of *FBN1* forms a glucogenic and appetite stimulating hormone termed asprosin (Romere et al. 2016), consistent with the phenotype of affected individuals.

Since its discovery (Romere et al. 2016) there has been considerable interest in this peptide hormone. The primary focus has been on the presence of elevated asprosin and implications for the pathology of metabolic syndrome (reviewed by Summers et al. 2023), but models of asprosin deficiency may also be beneficial to human patients who lack the ability to make asprosin because of a genetic variant. A mouse model was created with a small deletion encompassing the exon 65-intron 65 junction which leads to truncation of the profibrillin-1 molecule and loss of asprosin (Duerrschmid et al. 2017) (NPS; Table 2). Heterozygous mice replicated the hypophagia and reduced adiposity of the human cases. They were resistant to diet-induced obesity and diabetes. Reduced energy expenditure could be overcome by placing the animals on a high fat diet, which may be relevant to treatment of human patients with this condition. Also important for human therapy, a single dose of recombinant asprosin could rescue hypophagia (Duerrschmid et al. 2017), suggesting that this treatment may help human patients to increase their body weight and normalize the adipose phenotype, which would have important psychosocial benefits for these individuals. This model was made on the C57BL/6 strain which was found previously to have the lowest expression of *Fbn1* and adiposity of tested mouse strains (Davis et al. 2016) and the results need to be validated on other mouse strains.

Rabbits have been used extensively for research into cardiovascular and ocular conditions (Getz and Reardon 2012; Zernii et al. 2016). Rabbits with various deletions of the C-terminus of profibrilliln-1 were created using CRISPR-Cas9 technology (Chen et al. 2018) to examine the impact of asprosin deficiency. F1 heterozygous rabbits with a frameshift deletion showed the phenotype of Marfanoid-progeriod-lipodystrophy syndrome (Table 1) including absence of fat, reduced body weight and growth retardation as well as apparent progeroid facial features, slender limbs, aortic root dilatation, ocular, and pulmonary abnormalities. They also had poor survival, with death before age 25 weeks probably due to lung and/or aortic abnormalities. This phenotype was more severe than that seen in the asprosin-deficient mouse and comparable to the more extreme human Marfanoid-progeriod-lipodystrophy cases. Homozygous mutant rabbits could not be produced due to the high mortality of the heterozygous animals. Although the heterozygous rabbits recapitulate many of the signs of MFS and Marfanoid-progeriod-lipodystrophy, their utility as models is limited by the high mortality.

The phenotype of a spontaneous variant in Japanese Black cattle (Hirano et al. 2012) was described as “MFS-like”. The variant, c.8227-1G > A, affects the splice acceptor site of intron 64 in the bovine gene, which results in a frameshift that truncates the protein by 125 amino acids (p.Asn2743IlefsX5), encompassing most of the 140 amino acid asprosin sequence. The variant position corresponds to intron 65 in humans, distal to the critical furin cleavage site (Summers et al. 2023). Affected offspring were defined by having lower body weight with normal or increased withers height. This is consistent with reduced level of asprosin resulting in low body mass index in humans (Romere et al. 2016). These animals had narrow trunk width and long thin legs with long phalanx proximalis, similar to the tall thin body habitus of many human MFS and asprosin-deficient patients (Davis et al. 2016; Romere et al. 2016; Summers et al. 2023). They also had ocular abnormalities although ectopia lentis was not seen. A minor cardiovascular phenotype (pause between heart sounds) was also reported. Thus the phenotype is not as severe as the Limousin cross breed animals discussed above or as many human patients and resembles the human asprosin deficiency syndrome. Like the Limousin cross breed animals, these animals had a phenotypically normal sire who produced at least 36 affected offspring. Approximately 15% of the sperm of the sire carried the variant, confirming germline mosaicism. This cattle variant could be useful for studies of asprosin function in metabolic syndrome (Summers et al. 2023) as well as the lipodystrophy associated with MFS and Marfanoid-progeriod-lipodystrophy syndrome (Table 1).

## Models of other fibrillin-1 deficiency syndromes

While the majority of pathogenic variants in *FBN1* result in the phenotypes of MFS, there are also paradoxical phenotypes that have opposite signs and symptoms, including short stature and thick tight skin (Table 1), leading to the classification of *FBN1* variants as “tall” and “short” (Charbonneau et al. 2004; Peeters et al. 2022), reflecting the differences in skeletal phenotype.

Weill-Marchesani syndrome (WMS) involves short stature, brachydactyly, joint stiffness, and lens abnormalities (Faivre et al. 2003a). WMS can result from variants in *FBN1* (WMS2) or other genes including *ADAMTS10* (WMS1)*, LTBP2* (WMS3), and *ADAMTS17* (WMS4) (Faivre et al. 2003b; Hubmacher and Apte 2011). A mouse model for WMS2 (named WMΔ) was based on the deletion of exons 10–12 found in a human family with the syndrome (Table 2) (Sengle et al. 2012). The heterozygote replicated the thick skin, brachydactyly, and reduction of long bone growth in early life. The homozygote had a more severe phenotype but normal viability and neither heterozygotes nor homozygotes showed aortic root abnormalities or early death consistent with MFS. The results implicate interactions with members of the ADAMTS (a disintegrin and metalloproteinase with thrombospondin motifs) family, mutations in some members of which are also associated with WMS (Hubmacher and Apte 2011). However, these studies have not led to treatment for WMS in humans, which is currently focussed on management of visual and systemic manifestations (https://eyewiki.aao.org/Weill-Marchesani_Syndrome).

Stiff or tight skin is a feature of a number of *FBN1* variants in humans, and there are mouse models for some of these. Tight skin (*Tsk*) is natural variant in the mouse which results from a large in frame duplication within *Fbn1* (Siracusa et al. 1996). The main phenotypic characteristics are thick nonelastic skin and increased skeletal size. Homozygotes die in embryonic life but heterozygotes are viable with a normal life span (Green et al. 1976). This has been proposed as a model for human scleroderma and other conditions involving pathogenic tissue fibrosis. A similar phenotype, stiff-skin syndrome (SSS), maps to *FBN1* in humans (Table 1) and involves thickened skin over the whole body with limitations of joint mobility (Esterly and Mckusick 1971). Mouse models for 2 missense variants have been created (Table 2) (Gerber et al. 2013). Both replicate the aggressive skin fibrosis seen in human families with equivalent variants. There was also skin infiltration with proinflammatory immune cells. In the mouse models, the phenotype was resolved with integrin-modulating therapies or TGFB antagonism (Gerber et al. 2013) but these have shown limited success in human patients (https://www.uptodate.com/contents/stiff-skin-syndrome) where the sclerosis is noninflammatory (Liu et al. 2008).

## Fibrillin-2 phenotypes and models

Fibrillin-2 (*FBN2* gene, human chromosome 5) is the second member of the fibrillin family and is also implicated in the structure of connective tissue (Zhang et al. 1994; Putnam et al. 1995; Carta et al. 2006; Callewaert et al. 2009; Frederic et al. 2009). Fibrillin-2 is primarily synthesized in early life and is largely replaced by fibrillin-1 in adult humans in tissues and the ciliary zonule (Mariencheck et al. 1995; Charbonneau et al. 2010b; Jones et al. 2019). Fibrillin-2 has thus generally been seen as a protein required during development while fibrillin-1 is needed in adults to maintain homeostasis of connective tissues (Zhang et al. 1994; Sabatier et al. 2011). During postnatal development, fibrillin-2 may be present in the 10–12 nm microfibrils but it is increasingly masked by fibrillin-1 (Charbonneau et al. 2010b). In humans, expression of *FBN2* is largely confined to placenta, fibroblasts in culture, and embryonic mesenchymal cells while in mouse *Fbn2* expression is seen in umbilical cord and embryonic tissues. In sheep highest expression is seen in adult reproductive tissues, embryo, and some newborn samples while in the pig it is also highly expressed in some adult muscles (Table 3).

**Table 3.** Expression of fibrillin-2 and fibrillin-3 genes in mammalian transcriptomic atlases.

Species	Highest mRNA expression	Data sources
**Fibrillin-2**		
Human	Placenta Cultured fibroblasts Embryonic mesenchymal cells	http://biogps.org/#goto=genereport&id=2201; https://fantom.gsc.riken.jp/zenbu/gLyphs/#config=FANTOM5_promoterome_hg38;loc=hg38::chr5:128187825..128608329+
Mouse	Umbilical cord Embryonic tissues	http://biogps.org/#goto=genereport&id=14119; https://fantom.gsc.riken.jp/zenbu/gLyphs/#config=DmO_zYuHPxvu83HbOzew1C;loc=mm9::chr18:58117950..58419905+
Rat	Some adult brain regions Spinal cord Rib Some fetal tissues Adult reproductive tissues Embryo	http://biogps.org/ratatlas/#goto=genereport&id=689008
Sheep	Adult reproductive tissues Adult heart valves Embryo Some newborn tissues	http://biogps.org/sheepatlas/#goto=genereport&id=101104991
Pig	Embryo Some adult brain regions Some adult muscles	http://biogps.org/pigatlas/#goto=genereport&id=100048956
**Fibrillin-3**		
Human	Embryonic stem cells Fibroblasts Other stem cells Some fetal tissues	http://biogps.org/#goto=genereport&id=84467 https://fantom.gsc.riken.jp/zenbu/gLyphs/#config=FANTOM5_promoterome_hg38;loc=hg38::chr19:8044354..8170640+
Sheep	Embryo Embryonic ovary Embryonic brain Adult liver Adult cerebellum	http://biogps.org/sheepatlas/#goto=genereport&id=101103811
Pig	Embryo Forebrain Some adult muscle Adult female uterus Primordia of developing teeth	http://biogps.org/pigatlas/#goto=genereport&id=100738368

Like profibrillin-1, profibrillin-2 apparently generates a C-terminal fragment that acts as a glucogenic hormone, now called placensin (Yu et al. 2020) that is produced by placenta. There are no models for deficiency of this hormone.

### Congenital contractural arachnodactyly

Mutations in *FBN2* result in a phenotype that is largely musculoskeletal in humans. The cardinal features are contractures of the digits and folds of the pinnae of the ears (Hecht and Beals 1972) (Table 1) and the condition has been termed congenital contractural arachnodactyly (CCA), referencing the long spider-like fingers. Occasionally there may be cardiovascular signs and, rarely, aortic dissection (Gupta et al. 2002; Takeda et al. 2015; Milewicz and Cecchi 2023). The phenotype associated with *FBN2* variants is dominant and generally less severe than that associated with *FBN1* variants.

Mouse models do not faithfully replicate the human phenotype (Table 4). A null allele for *Fbn2* resulted in a homozygous phenotype said to recapitulate human CCA but the mice also showed syndactyly (Arteaga-Solis et al. 2001). Syndactyly is not a feature of CCA caused by *FBN2* variants in humans. Recently, CRISPR-Cas9 targeted mutation (em1(IMPC)Rbrc) created a multisystem phenotype (Table 4). There is little information about this allele and it is not clear whether some aspects of the phenotype may result from off-target effects. These *Fbn2* alleles tend to be associated with more extensive phenotypes than generally seen in humans, possibly due to the lack of fibrillin-3 (see The problem of fibrillin-3 section). In addition, all are inherited in a recessive manner, which is different from CCA in humans. Sy is a radiation-induced deletion in mouse that involves 20 genes including *Fbn2*. It results in syndactyly and auditory/vestibular abnormalities (Grüneberg 1962; Johnson et al. 1998). It is early lethal in homozygotes. Allelic spontaneous mutations of the same region also caused syndactyly or hearing loss (Johnson et al. 1998). Subsequent studies of the allelic series showed that the auditory defects were attributable to deletion of *Slc12a2*, encoding a sodium/potassium/chloride cotransporter (Dixon et al. 1999), while the syndactyly resulted from variants of *Fbn2* (Chaudhry et al. 2001).

**Table 4.** Mouse models of fibrillin-2 syndromes.

Model name	Variant type	Strain	Phenotype	Inheritance	Syndrome	Reference
tm1Rmz	ins PGK*neo* replacing 1.2 kb of exon 1	129/Sv C57BL/6J × 129/Sv	Contractures of carpal, metacarpal and phalangeal joints, stiff hindlimbs, bilateral syndactyly	recessive	CCA + syndactyly; distal arthrogryposis	Arteaga-Solis et al. 2001
timon	del 1.7 kb including exon 63	C57BL/6J	Wrist contracture, micrognathia, eye and ear defects, hindlimb syndactyly	recessive		Geister et al. 2018
mz	p.Leu621ter	BALB/c × C3H/HeH	Poor muscle strength, hindlimb syndactyly, reduced growth rate	recessive	Distal arthrogryposis	Miller et al. 2010
em1(IMPC)Rbrc	CRISPR-Cas9 mediated indel in exon 6*^a^*	C57BL/6NJcl	Abnormal skeletal, joint, eye, heart morphology, syndactyly, reduced grip strength	recessive		https://www.mousephenotype.org/data/genes/MGI:95490
sy	del ~2 mb, 20 genes	B6C3Fe	Fore- and hindlimb syndactyly	dominant		Johnson et al. 1998; Chaudhry et al. 2001

List derived from the Mouse Genome Informatics (MGI) database (https://www.informatics.jax.org/allele/summary? markerId=MGI:95490) and relevant publications. A number of conditional mutations in the *Fbn2* locus are listed on the MGI website; these are not included here as they do not replicate the human situation where the variant is present in all cells and tissues.

del, deletion; ins, insertion; dup, duplication; indel, small insertion or deletion (unspecified); CCA, congenital contractural arachnodactyly; kb, kilobases; mb, megabases.

^*a*^Exon determined by Ensembl BLASTN search (http://www.ensembl.org, accessed August 2023) of mouse reference genome using published guide sequence CCCTGGCACATCTGATTATTGAC (https://www.informatics.jax.org).

### Bicuspid aortic valve

Bicuspid aortic valve is a congenital malformation with a prevalence of about 2% in humans and lifetime risk of complications in those with a bicuspid aortic valve (including aortic dilatation and dissection) of around 30% (Bravo-Jaimes and Prakash 2020). Heritability is as high as 90% (Loscalzo et al. 2007), but even within families, it shows incomplete penetrance and variable expressivity, indicating that a number of interacting genes may be involved. Most cases are sporadic (Michelena et al. 2021). It is occasionally seen in patients with MFS (Braverman and Roman 2019) though the relationship to the *FBN1* variant has not been established. More commonly it is seen in patients with Loeys-Dietz syndrome, where TGFB signaling is disrupted, which has a phenotype that overlaps with MFS (Chaudhry et al. 2007; Michelena et al. 2021). *FBN1* variants have been seen in patients with bicuspid aortic valve without other features of a fibrillinopathy, but the significance of this finding is unclear (Pepe et al. 2014; Girdauskas et al. 2017).

Genetic variants in *FBN2* were not assessed in the previous human studies (Pepe et al. 2014; Girdauskas et al. 2017). However, the aortas of humans with bicuspid aortic valves were found to have elevated levels of *FBN2* mRNA (Rueda-Martinez et al. 2017). In the Syrian hamster, 1 inbred strain is prone to bicuspid aortic valve with an incidence of around 40% (Fernandez et al. 2020; Soto-Navarrete et al. 2022). *Fbn2* mRNA was increased in the aortas of these susceptible animals, particularly in those with a bicuspid valve (Soto-Navarrete et al. 2022). There are a number of genetic models in the mouse for bicuspid aortic valve; most affect transcription factors and none implicate *Fbn2* or TGFB signaling directly (Fernandez et al. 2020). The potential involvement of fibrillin-2 in the etiology of bicuspid aortic valve suggests that it would be valuable to assess the mouse strains with *Fbn2* variants for formation and function of the aortic valve, and to assess the mouse strains with bicuspid aortic valves for abnormalities in *Fbn2* expression and fibrillin-2 protein. In addition, the creation of strains overexpressing *Fbn2* might provide a model for the increased expression of *FBN2* seen in human patients and hamsters and help to unravel the role of fibrillins and TGFB signaling in bicuspid aortic valve.

## The problem of fibrillin-3

The final member of the fibrillin family is fibrillin-3 (*FBN3* gene, human chromosome 19) (Corson et al. 2004). The mRNA for this protein is found mainly in female reproductive and some fetal and embryonic tissues and pluripotent cell lines (Table 3, Supplementary Table 1). The Ensembl database (http://www.ensembl.org, accessed August 2023) currently lists 96 species with a potential fibrillin-3 gene in a region syntenic with the human *FBN3* gene region. In mice and rats the ancestral fibrillin-3 gene appears to have a large central deletion and multiple in frame stop codons so is likely to be nonfunctional (Nagase et al. 2001; Corson et al. 2004) and in guinea pigs the syntenic sequence apparently encodes only the C-terminal half of the protein and includes a nonhomologous insertion compared to the human sequence (http://www.ensembl.org, accessed August 2023) (Piha-Gossack et al. 2012). As there is no equivalent functional gene in rodents (Nagase et al. 2001; Corson et al. 2004; Piha-Gossack et al. 2012), unlike all other mammals including rabbits and 2 species of squirrel, conventional mouse and rat models cannot be made. This means that research on fibrillin-3 has been limited by a lack of convenient model species.

Studies in humans and bovines (Urbanek et al. 2007; Prodoehl et al. 2009a, 2009b; Jordan et al. 2010; Azumah et al. 2022) suggest that the protein is important in female reproductive organs and in gametes (Table 3). Genome-wide association studies (GWAS) identified a locus for polycystic ovary disease that encompassed the human *FBN3* gene (Urbanek et al. 2007; Prodoehl et al. 2009a). *FBN3* protein was reduced in polycystic ovaries when compared with normal ovaries (Jordan et al. 2010). Results suggested that fibrillin-3 functions in the transition from primordial follicle to primary follicle. In addition, *FBN3* mRNA level decreased across gestation in the fetal bovine ovary, consistent with a role in early development of female reproductive organs (Azumah et al. 2022) (Table 1).

The absence of fibrillin-3 in rats and mice raises 2 issues. First, as mentioned above, there are no models for deficiency of this protein. As no human single gene phenotype has been reported, either absence of fibrillin-3 is lethal early in development, or redundancy with the 2 other fibrillins allows normal development without fibrillin-3. It is not possible to test this in mouse and rat models but gene editing of large animals is now well established (Whitelaw et al. 2016) and knock-out of the *FBN3* gene could be achieved. As the protein may play a role in fecundity and fertility of production animals (Azumah et al. 2022), it seems targeting *FBN3* could be informative for improving food sustainability as well as increasing understanding of the fibrillinopathies.

Polycystic ovary disease is one risk factor associated with metabolic syndrome in humans. Given the role of the terminal peptides from fibrillin-1 (asprosin) and fibrillin-2 (placensin) in glucose metabolism it seems possible that a similar peptide from the end of fibrillin-3 might be important in glucose homeostasis of the ovary, developing oocytes and zygote (Summers et al. 2023). An animal model where *FBN3* could be manipulated would assist in examining this hypothesis. As discussed further below, the chicken has a functioning *FBN3* gene (Corson et al. 2004) and has lines extensively bred for reproduction. This may lead to a relevant model for *FBN3* deficiency.

The second problem with the lack of fibrillin-3 in mice and rats is that it questions the relevance of the mouse models of fibrillin-1 and fibrillin-2 deficiency. It seems likely that fibrillin-2 takes over the role of fibrillin-3 in these species (Davis et al. 2014) which may account for the more severe phenotype of the *Fbn2* genetically modified mice compared to *FBN2* variants in humans. A model in a similar species that has a functional *FBN3* gene would help clarify the impact of lacking the gene in mice and rats and hence the relevance of these model animals. The rabbit has an annotated full length *FBN3* gene in the region syntenic to the human *FBN3* gene region (ENSOCUG00000011511; http://www.ensembl.org, accessed August 2023). Since the rabbit has been amenable to genome modifications (Chen et al. 2018; Song et al. 2020) this would be a worthwhile species with which to explore fibrillin-3 variation.

## Other potential models for fibrillinopathies

While the majority of animal models for human genetic disease are created or arise spontaneously in mice, other species may also carry mutations equivalent to human variants, as indicated above for the bovine *FBN1* variants. The fibrillins are highly conserved across species from cnidarians to mammals (Piha-Gossack et al. 2012) (Table 5). This section explores possible models in other species.

**Table 5.** Annotated fibrillin genes in representative non-Mammalian eukaryotes.

Type	Species	Gene symbol	Ensembl ID	Position	Syntenic human gene*^a^*
Bird	*Gallus gallus* (Chicken reference)	*FBN1*	ENSGALG00010018446	10: 10,196,204–10,347,306	*FBN1*
		*FBN2*	ENSGALG00010012940	Z: 57,572,115–57,735,283	*FBN2*
		Novel gene	ENSGALG00010028301	28: 748,104–807,082	*FBN3*
	*Taeniopygia guttata* (Zebra finch)	*FBN1*	ENSTGUG00000019967	10: 10,687,753–10,833,054	*FBN1*
		*FBN2*	ENSTGUG00000006641	Z: 19,412,687–19,528,936	*FBN2*
		Novel gene fragments*^b^*	ENSTGUG00000000946 ENSTGUG00000022997 ENSTGUG00000023585 ENSTGUG00000025566	28:1,198,218–1,257,990	*FBN3*
Reptile	*Pelodiscus sinensis* (Chinese soft shell turtle)	*FBN1*	ENSPSIG00000013146	Scaffold JH212494.1: 1,344,550–1,567,396	*FBN1*
		*FBN2*	ENSPSIG00000014580	Scaffold JH211314.1: 448,230–733,300	*FBN2*
		Novel gene*^c^*	ENSPSIG00000014464	Scaffold JH209348.1: 425,806–425,933	*FBN3*
	*Crocodylus porosus* (Australian salt water crocodile)	*FBN1*	ENSCPRG00005014181	MDVP01000032.1: 22,496,653–22,704,014	*FBN1*
		*FBN2*	ENSCPRG00005010570	MDVP01000068.1: 10,977,784–11,261,323	*FBN2*
		*FBN3*	ENSCPRG00005013427	MDVP01000063.1: 4,384,393–4,473,954	*FBN3*
Amphibian	*Xenopus tropicalis* (Clawed toad)	*fbn1*	ENSXETG00000008779	3: 103,789,676–103,911,959	*FBN1*
		*fbn2*	ENSXETG00000032535	1: 167,581,240–167,726,912	*FBN2*
		*fbn3*	ENSXETG00000001781	1: 92,395,786–92,500,369	*FBN3*
Fish*^d^*	*Takifugu rubripes* (Fugu)	*fbn2b*	ENSTRUG00000012996	20: 9,649,309–9,687,408	*FBN3*
	*Danio rerio* (Zebrafish)	*fbn1*	ZDB-GENE-091204–466*^e^*	18:5,316,207–5,345,282	*FBN1*
		*fbn2a*	ENSDARG00000051896	10:16,313,851–16,474,216	*FBN2*
		*fbn2b*	ENSDARG00000098237	22:4,524,367–4,649,238	*FBN3*
	*Gadus morhua* (Atlantic cod)	*FBN1*	ENSGMOG00000008510	14: 13,478,893–13,542,240	*FBN1*
		*fbn2b*	ENSGMOG00000009755	12: 26,359,137–26,397,080	[*FBN3*]*^f^*
	*Salmo salar* (Atlantic salmon)	*FBN1*	ENSSSAG00000097601	11: 32,338,996–32,703,955	*FBN1*
		*FBN1*	ENSSSAG00000095019	26:32,713,409–32,886,730	*FBN1*
		*fbn2a*	NSSSAG00000077250	1:166,160,786–166,373,162	*FBN2*
		*fbn2b*	ENSSSAG00000007909	10:19,346,980–19,436,215	*FBN3*
		*fbn2b*	ENSSSAG00000057875	16:39,779,019–39,904,486	
	*Scleropages formosus* (Asian bonytongue)	*FBN1*	ENSSFOG00015017770	7: 31,324,447–31,376,124	*FBN1*
		*FBN1*	ENSSFOG00015013164	11: 11,118,508–11,169,624	*FBN1*
		*fbn2a*	ENSSFOG00015007953	6: 13,440,703–13,514,547	*FBN2*
		*fbn2b*	ENSSFOG00015006450	9: 832,842–881,796	*FBN3*
Insect	*Apis mellifera* (Honey bee)	*LOC409950*		CM009932.2:639479–663432	*FBN1*
		*LOC100577456*		CM009941.2:11397504–11424515	*FBN2*
		*LOC724421*		CM009945.2:5255226–5265494	*FBN2*
		*LOC725800*		CM009936.2:4978223–5138349	*FBN2*
Nematode	*Caenorhabditis elegans*	*fbn-1*	WBGene00022816	Chromosome III: 7,625,386–7,641,078	fibrillins
		*mua-3*	WBGene00003482	Chromosome III: 10,160,697–10,183,529	fibrillins

Gene annotations and positions taken from Ensembl (Release 110, July 2023; http://www.ensembl.org).

^*a*^Synteny based on presence of at least 2 of the same annotated flanking genes in both species.

^*b*^Detected by performing TBLASTN search in Ensembl using the protein sequence derived from ENSGALG00010028301.

^*c*^Detected by performing TBLASTN search in Ensembl using the protein sequence derived from ENSCPRG00005013427.

^*d*^Whole genome duplication occurred in the ancestor of teleost fishes. Some lineages including the salmonids have undergone subsequent additional whole genome duplication. There has also been extensive genome rearrangement during evolution of the fish lineages, which means that the synteny with the human genome often breaks down. There may eventually have been loss of 1 member of the duplicated gene pair, both pairs may have retained the same function, the function may have been divided between the 2 pairs or 1 pair may have acquired a new function. It is likely that the ancestral teleost had 3 distinct fibrillin genes, corresponding to human *FBN1, FBN2*, and *FBN3*. In most species at least 80% of the duplicated genes have subsequently been lost; for the salmonids up to 50% have been retained. This is consistent with the different numbers of fibrillin genes found in fugu and zebrafish when compared with Atlantic salmon. See Glasauer and Neuhauss (2014) for a review. It is also likely that annotation of the homologs and paralogues has been difficult in the fish sequences because of the multiple copies present.

^*e*^ *fbn1* is not annotated in the Ensembl and University of California Santa Cruz (UCSC) browsers but has been annotated in the NCBI Gene database in a position syntenic with human *FBN1*. The ID given here is the Zebrafish Information Network (ZFIN) accession.

^*f*^Only 1 matching flanking gene; many unannotated genes in region.

### Other mammalian models

Rats are evolutionarily closer to humans than mice (Pradhan and Majumdar 2016) and have been used since the early 20th century for biomedical research (Gibbs et al. 2004). The Rat Genome Database (Kaldunski et al. 2023) indicates that there are several relevant quantitative trait loci (QTL) in the *Fbn1* region, including QTL for body weight, cardiac mass, epididymal fat weight, and vascular elastic tissue fragility, all relevant to fibrillinopathies. Given that fibrillin-1 is involved in determining adipose level in human and mouse (Davis et al. 2016) and that deficiency is associated with major abnormalities of the walls of the large arteries (Loeys et al. 2010), these QTL should be further explored. While rats provide the dominant laboratory models for cardiovascular and metabolic conditions (Gibbs et al. 2004), there is no published rat model for MFS or related conditions. Recently modification of the rat genome has become more common (Pradhan and Majumdar 2016; Meek et al. 2017; Nohmi et al. 2017), and creation of rat models of fibrillinopathies would be feasible. It would be advantageous to have a rat model since the larger size of the rat and availability of strains with known cardiovascular phenotypes would open opportunities for experimentation and development of treatment. An asprosin-deficient rat strain would be particularly helpful and could be combined with the known metabolic models (such as the spontaneously hypertensive, Dahl salt sensitive hypertensive and Zucker diabetic strains and obesogenic protocols) to explore the role of asprosin and fibrillin in metabolic syndrome.

Dogs (and to a lesser extent cats) have been found to segregate pathogenic variants in genes that also cause disease in humans (Parker et al. 2004; Ostrander and Wayne 2005; Parker and Ostrander 2005; Lyons 2010; Hytönen and Lohi 2016) (see also Online Mendelian Inheritance in Animals, https://www.omia.org/home/). While there is no canine breed that segregates a Marfan-like condition, the fibrillins may be involved in variance in size, body weight, and relative limb length. Several breeds are prone to cardiovascular conditions, particularly giant breeds such as the Leonberger, Great Dane, and Irish Wolfhound (Shen et al. 2022). Dogs also frequently suffer ocular conditions including lens subluxation, also seen in cats (Gould et al. 2010; Payen et al. 2011). Examination of *FBN1* variation in these domestic species may yield further models for MFS and its complications. The enrollment of client-owned animals in genetic studies has been a productive and economical way of exploring familial conditions, particularly where a condition is limited to a specific breed and the pedigree of animals is well known (Hytönen and Lohi 2016; Schulte and Arlt 2022).

### Models in other vertebrates

Genome editing is now possible in the chicken (Idoko-Akoh and Mcgrew 2023). Avian embryos are easily accessible for developmental studies and have been used to observe the distribution of fibrillin-1 during early development (Burke et al. 2000). It is possible that fibrillin-1 abnormalities contribute to myopathic phenotypes in broiler chickens (Bordini et al. 2021). A dietary model of homocysteinemia in the chick showed disruption of fibrillin-2 fibers associated with high plasma methionine, and fibrillin-2 deposition has been extensively studied in the Japanese quail (*Coturnix japonica*) (Rongish et al. 1998). As suggested above, chickens could also be used to examine the functions of fibrillin-3, bearing in mind the different reproductive models.

Extensive divergent selection for meat (broilers) or eggs (layers) has impacted muscle, bone, and fat production in chicken lines (Gheyas et al. 2015; Boschiero et al. 2018). These are tissues with high expression of fibrillins, and comparisons between inbred lines with extreme phenotypes may reveal functions of the fibrillins which could be translated to humans. The chicken reference genome shows a number of potentially damaging variants in chicken fibrillin genes (Table 6). Examination of the phenotype of animals carrying these variants may provide useful chicken models for various fibrillinopathies.

**Table 6.** Variants in fibrillin genes of chicken (*Gallus gallus*).

Gene	Total missense variants	Missense variants predicted deleterious*^a^*	Number of stop gained variants
*FBN1*	27	20	2
*FBN2*	8	6	1
*FBN3*	84	75	1

Derived from http://www.ensemble.org, accessed August 2023. Full list is available in Supplementary Table 1.

^*a*^Predicted by the Sorting Intolerant from Tolerant (SIFT) algorithm (Ng and Henikoff 2003).

The Japanese quail has been a popular model for research in genetics, developmental biology, behavior, and biomedical studies. A high-quality genome of the quail is now available (Morris et al. 2020). The Ensembl genome browser does not show any fibrillin gene variants in the quail, but there is ongoing research, including experiments with hybrids between chicken and quail (Zhu et al. 2021). This species may therefore provide a tractable model in the future.

The zebrafish (*Danio rerio*) has increased in popularity as a model for human genetic diseases. The status of the *fbn1* gene in this species is uncertain. Neither Ensembl nor UCSC browser shows a gene encoding fibrillin-1 in the chromosome region syntenic with the mammalian *FBN1* region and there are no annotated transcripts or expressed sequence tags mapped to the region in either browser. The syntenic region contains a sequence with limited homology to human *FBN1* and this has been annotated as *fbn1* in the NCBI database (https://www.ncbi.nlm.nih.gov/gene/100330961). There is very little similarity between the amino acid sequence encoded by this gene and the human or chicken fibrillin-1 (Ensembl BLASTN search using XP_017207479.2, August 2023) with only a small number of stretches of around 40 amino acids showing a match at >50% identity. It is not clear whether this sequence represents a functional gene in zebrafish. However, 2 studies have proposed zebrafish as a model for fibrillin-1 deficiency in humans. A heterozygous frameshift insertion/deletion mutation in this putative zebrafish *fbn1* produced a phenotype with some features in common with MFS (Yin et al. 2021). A different frameshift insertion/deletion mutation was used to examine ocular development with a heterozygous phenotype that showed some features of MFS (Quint et al. 2022). The zebrafish retains 2 other intact fibrillin genes, which are in regions syntenic with human *FBN2* (*fbn2*) and *FBN3* (*fbn2b*) (Table 5). Valuable insights into the role and function of fibrillins may be gained using genetic manipulation of the zebrafish genome although it may not act as a true model for the human deficiency syndromes.

### Nonvertebrate models

Models for fibrillinopathy have also been proposed in nonvertebrate species. These could provide useful understanding of the roles of the fibrillins, while not precisely modeling the conditions as presented in humans.

A model organism that has allowed considerable understanding of development and disease is the nematode worm *Caenorhabditis elegans* (https://www.ncbi.nlm.nih.gov/books/NBK20086/) (Culetto and Sattelle 2000). The *C. elegans* genome is thought to contain 2 fibrillin homologs, *fbn-1* and *mua-3*. These show a maximum identity of 45% (*mua-3* isoform a and *fbn-1* isoform h) and 32% (*mua-3* isoform b) (Bercher et al. 2001; Frand et al. 2005) with human fibrillin-1. These genes are believed to affect *C. elegans* tissue equivalent to connective tissue in higher eukaryotes (Bercher et al. 2001; Frand et al. 2005). A proposed *C. elegans* model for MFS has been developed (Fotopoulos et al. 2015) in which a temperature sensitive deletion allele of *mua-3* was shown to interact with a TGFB2 homolog as well as a collagen. Based on their results, the authors propose that metabolic rate might be a factor in the phenotype of MFS and that a drug that reduced metabolic rate might be appropriate (Fotopoulos et al. 2015). This is interesting in the context of the role of asprosin in metabolism. However, there has been no follow up of this suggestion, and it is unknown whether either of the *C. elegans* genes can be cleaved to produce a similar peptide. In addition, the encoded *C. elegans* proteins lack the typical fibrillin TB and hybrid domains and cannot be considered true fibrillin homologs (Piha-Gossack et al. 2012), suggesting that these findings may have limited application to human fibrillinopathies.

A search for “fibrillin” in Ensembl metazoa (http://metazoa.ensembl.org/) yielded 987 genes described as fibrillin-1, fibrillin-2, fibrillin-3, fibrillin-1-like, fibrillin-2-like fibrillin-3-like, or putative fibrillin. The list of invertebrate species with an annotated fibrillin gene includes several of economic (various bee species), medical (ticks and mosquitoes), or ecological (coral polyps) importance. Exploration of fibrillins in these invertebrate species could, therefore, bring benefits beyond modeling human genetic diseases. However, these species are not particularly amenable to the creation of models and further studies may rely on the discovery of natural variants. The popular laboratory insect *Drosophila melanogaster* did not have a homologous sequence (Piha-Gossack et al. 2012) (http://www.ensembl.org/ and http://flybase.org/; accessed August 2023), meaning that this model animal has little relevance to the study of fibrillinopathies.

### Cell culture models

Cell culture models have been promoted as an alternative to expensive animal models with their associated welfare and ethical issues. Fibrillin mRNA expression in a range of cultured human primary cells and cell lines is shown in Supplementary Table 2. There is negligible expression of any fibrillin gene in cells of the immune system, hepatocytes, or neurons but high expression of *FBN1* and *FBN2* in cells of mesenchymal origin, particularly those in a relatively undifferentiated state such as fibroblasts, preadipocytes, and de-differentiated chondrocytes. *FBN3* mRNA expression is much lower than *FBN1* and *FBN2* and is largely found in cells of embryonic origin and in induced pluripotent stem cells. Some examples where in vitro studies have given insights into the function of fibrillins and the nature of fibrillinopathies are given below, indicating that cells in culture can provide a range of models.

Initial understanding of the structure of the extracellular matrix and the role of fibrillins was obtained using cultured fibroblasts explanted from patient skin or purchased from cultured cell repositories. For example, early studies showed that cultured fibroblasts from patients with MFS lack the extracellular microfibrils characteristic of normal fibroblasts and replicated the histology of skin biopsies (Godfrey et al. 1990; Hollister et al. 1990). Since then cultured fibroblasts have been used to demonstrate the processing of fibrillin-1 (Milewicz et al. 1992; Aoyama et al. 1994). Cultured aortic vascular smooth muscle cells were used to explore extracellular matrix abnormalities associated with MFS and bicuspid aortic valve (Nataatmadja et al. 2003). Fibroblasts from a patient with geleophysic dysplasia due to a variant in FBN1 were used to test the effect of losartan treatment and results suggested that this would be a promising treatment for these patients (Piccolo et al. 2019). *FBN2* is also expressed by fibroblasts and its role in the response to hypoxia has been investigated in fibroblast cultures (Boizot et al. 2022). Human fibroblasts purchased from a cell bank have been used to demonstrate that both *FBN1* and *FBN2* mRNA levels increased in response to administration of human ceramides (Sugahara et al. 2022).

In vitro transdifferentiation of fibroblasts to vascular smooth muscle cell morphology was used to show that cells with a pathogenic *FBN1* variant had reduced transdifferentiation efficiency, with a reduction in smooth muscle actin fibers (Burger et al. 2021). This could have implications for the formation of aortic aneurysms, which result from failure of the extracellular matrix around aortic smooth muscle cells, and may provide a model to ascertain the impact of variants of unknown significance as well as testing the appropriateness of drug treatments.

Since many fibrilliinopathies have significant bone-related pathology, osteosarcoma cell lines which are derived from bone cancer may provide understanding of the timing and function of fibrillins in bone formation. For example, we have shown that the mature osteosarcoma line SAOS-2 (which is able to mineralize) does not express fibrillin-1 mRNA or protein but the early stage line MG63 (which fails to mineralize in vitro) expresses both (Summers et al. 2023). Similarly, 1 human chondrocyte line was able to form a fibrillin-1 matrix, while another, considered more mature, could not (Summers et al. 2023). Comparison of these different cell lines may show how expression of fibrillin-1 is regulated during formation and maturation of bone.

In several fibrillinopathies, development of adipose tissue is disrupted, possibly related to the absence of the terminal asprosin peptide (Romere et al. 2016) (See Models of Marfanoid-progeroid-lipodystrophy syndrome and asprosin deficiency section). Fibrillin-1 is expressed early in adipocyte differentiation but declines as the precursors become mature adipocytes (Davis et al. 2016). Further studies using these cells may assist not only in developing therapies for individuals with fibrillinopathies but also in understanding and treating metabolic syndrome.

Recently, induced pluripotent stem cell lines have been created from MFS patients (Li et al. 2019; Pan et al. 2021; Qin et al. 2021; Peeters et al. 2022; Yu et al. 2022). Since these cells can be differentiated in vitro into a variety of cell types, they may prove useful in exploring the impacts of fibrillin abnormalities across a spectrum of variants and tissues.

## Conclusions

Fibrillinopathies are among the more common Mendelian diseases in humans (Pyeritz 2019). They cause considerable morbidity and mortality and can be associated with psychosocial consequences beyond the pathophysiological problems. Models that replicate the various human fibrillinopathies would be valuable in understanding and developing treatments for these conditions.

To date, there is no model that shows all the features of human MFS. This may be due to the different nature of mouse compared with human and particularly the lack of fibrillin-3 in rodents, but large animal models (bovine, porcine) did not completely match the human condition either. The range of phenotypes resulting from *FBN1* variants means that many different models will be needed to completely cover the spectrum of sometimes conflicting manifestations (Table 1). The mouse models for *FBN2* deficiency appear to have a more severe phenotype than the human conditions and further development of these models is necessary. The lack of a rodent *Fbn3* means that researchers will have to be creative to find appropriate models to study this gene. This might include avian models which provide easy access for studying embryonic development. Although they do not replicate the environment of cells in in vivo, cells in culture have provided insights into the functions of the fibrillin proteins, and can be a valuable adjunct to animal and human studies.

Animal models are important in our attempts to understand human biology and pathology. They have limitations but even when a model fails to completely replicate the human disease, lessons can be taken from the model (Elsea and Lucas 2002). It is to be hoped that the search for reliable models of fibrillinopathies will persist and advances in understanding and treatment will continue to be made.

## Supplementary Material

iyad189_Supplementary_Data

## Data Availability

The data presented are from public databases. Supplemental material available at GENETICS online.
