# Supplementary material for: Genetic models of fibrillinopathies
Source: Genetics. 2023 Nov 16;226(1):iyad189. doi: 10.1093/genetics/iyad189 (PMC11021029; doi:10.1093/genetics/iyad189)
Supplement: iyad189_Supplementary_Data [file iyad189_supplementary_data.zip › Supplemental_Material_Legends_GENETICS-2023-306535.docx]

**SUPPLEMENTARY MATERIAL**

Table S1. Variants in fibrillin genes of the chicken (*Gallus gallus*)

Table S2. Expression of fibrillin genes in human primary cells and cell lines
